# Social effects of rabies infection in male vampire bats (*Desmodus rotundus*)

**DOI:** 10.1098/rsbl.2022.0298

**Published:** 2022-09-07

**Authors:** Elsa M. Cárdenas-Canales, Sebastian Stockmaier, Eleanor Cronin, Tonie E. Rocke, Jorge E. Osorio, Gerald G. Carter

**Affiliations:** ^1^ Department of Pathobiological Sciences, School of Veterinary Medicine, University of Wisconsin-Madison, Madison, WI 53706, USA; ^2^ Department of Evolution, Ecology and Organismal Biology, The Ohio State University, Columbus, OH 43210, USA; ^3^ U.S. Geological Survey, National Wildlife Health Center, Madison, WI 53711, USA; ^4^ Smithsonian Tropical Research Institute, Balboa Ancón, Panama

**Keywords:** disease ecology, social behaviour, pathogen manipulation, infection-induced behavioural changes, rabies virus

## Abstract

Rabies virus (RABV) transmitted by the common vampire bat (*Desmodus rotundus*) poses a threat to agricultural development and public health throughout the Neotropics. The ecology and evolution of rabies host–pathogen dynamics are influenced by two infection-induced behavioural changes. RABV-infected hosts often exhibit increased aggression which facilitates transmission, and rabies also leads to reduced activity and paralysis prior to death. Although several studies document rabies-induced behavioural changes in rodents and other dead-end hosts, surprisingly few studies have measured these changes in vampire bats, the key natural reservoir throughout Latin America. Taking advantage of an experiment designed to test an oral rabies vaccine in captive male vampire bats, we quantify for the first time, to our knowledge, how rabies affects allogrooming and aggressive behaviours in this species. Compared to non-rabid vampire bats, rabid individuals reduced their allogrooming prior to death, but we did not detect increases in aggression among bats. To put our results in context, we review what is known and what remains unclear about behavioural changes of rabid vampire bats (resumen en español, electronic supplementary material, S1).

## 1. Introduction

Rabies virus (RABV) transmitted by the blood-feeding common vampire bat (*Desmodus rotundus*) creates a substantial burden for agricultural development and public health throughout Latin America, with deadly rabies outbreaks occurring in livestock [1–3] and humans [4,5]. RABV is transmitted by direct contact between the virus-laden saliva of the infected bat and the other animal's broken skin, eyes or mucous membranes. RABV transmission can occur both among vampire bats and when they bite livestock, wildlife, or less frequently, humans, leading to cross-species transmission [2,6]. Recent studies combining mathematical modelling, RABV phylodynamics, and the ecology, demography, and dispersal of vampire bats have shown great potential to predict and mitigate these pathogen spillover events [6–9]. Surprisingly few studies, however, have directly investigated how RABV infection affects vampire bat behaviour.

In mustelids [10,11], canines [12,13], rodents [14], and humans [15], RABV can lead to paralysis without obvious increases in aggression before death (‘paralytic’ rabies), but it can also induce aggression and biting (‘furious’ rabies), which is likely to increase transmission to other hosts (pathogen manipulation [16]). Given that aggressive interactions are commonly observed in vampire bats, especially among males [17–19], and that RABV is detectable in the saliva at the end of infection [20,21], increases in aggression in rabid vampire bats could enhance transmission. In a previous study [21], seven confirmed naturally RABV-exposed vampire bats showed no obvious symptoms and survived, while seven others presented two distinct disease outcomes. Three bats showed *furious* rabies presentation with hypersalivation, excess vocalizations, teeth chattering, aggression towards handlers and other bats, and irritability to light and sound. Four bats showed *paralytic* rabies presentation with social isolation, lethargy, and apparent respiratory distress. Although these anecdotal observations demonstrate both presentations are possible, they appear in some studies but not others (table 1), and the relative probability of paralytic versus furious symptoms in rabid vampire bats remains unclear.

**Table 1.** Studies anecdotally describing rabies-induced changes in social behaviour of vampire bats after injection or natural exposure.

reference	result	method	type of virus used	comments
present study	no increase in aggressive behaviour observed, reduced social grooming	behavioural sampling	coyote variant	40 male vampire bats, some previously vaccinated, 15 bats where rabies confirmed
[22]	no signs of aggression reported, grooming unobserved	anecdotal observation	T-9/95 vampire bat field isolate from before 2001 (no detailed information). Study published in 2009. Isolate location unknown	10 vampire bats (eight males, two females); four bats confirmed positive; signs of paralytic rabies in all four
[23]	no signs of aggression reported, grooming unobserved	anecdotal observation	CASS-88 is of vampire bat origin and was isolated in 1988. Study published in 1998	24 vampire bats (no information on sex). Some bats were previously vaccinated with oral vaccine. Sixteen confirmed positive. Signs of paralytic rabies in 10 out of 16. Others showed no obvious clinical signs
[20]	no signs of aggression reported, grooming unobserved	anecdotal observation	CASS-88 is of vampire bat origin and was isolated in 1988. Study published in 2005	14 bats (six males, eight females), 11 died. Paralysis of wings and hind-legs prior to death in 3 out of 11; others showed depression, hypoactivity and anorexia
[24]	no signs of aggression reported, grooming unobserved	anecdotal observation	CASS-88 is of vampire bat origin and was isolated in 1988. Study published in 2002	test of rabies vaccine; 9 out of 10 control bats (injected with saline) died of rabies (no information on sex). Altered reflexes, tremor and paralysis were observed 72–24 h before death in rabid bats
[25]	no signs of aggression reported, grooming unobserved	anecdotal observation	Brldr2918 vampire bat field isolate from 1997. Study published in 2005. Study bat location and location of virus isolate are approximately 100 km apart	10 bats died of RABV (no detailed information on sex/deaths). Eight showed signs of paralytic rabies. Two showed no clinical signs
[26]	no signs of aggression reported, grooming unobserved	anecdotal observation	Brldr2918 vampire bat field isolate from 1997. Study published in 2008. No information on bat capture location	10 bats died of RABV (no detailed information on sex/deaths). Some were previously vaccinated (orally, applied to fur). All showed signs of paralytic rabies
[21]	aggression, grooming unobserved	anecdotal observation	natural exposure	total of 14 confirmed rabid male bats; seven showed no clinical signs: three the furious form and four the paralytic form
[27]	potential aggression, grooming unobserved	anecdotal observation	natural exposure	introduction of wild bats to an existing captive colony (no information on sex). After two months, fighting started. Several bats from original colony were mutilated and tested for rabies. Authors suggested aggression in introduced bats
[28]	aggression, grooming unobserved	anecdotal observation	natural exposure	aggression observed in naturally infected rabid bats; 14 of 24 bats observed showed clinical signs including hyperexcitability, aggressiveness, and paralysis before death

Besides biting, another possible transmission pathway is allogrooming, i.e. the licking and chewing of a conspecific's fur and skin [29]. Allogrooming takes up about 3–5% of a bat's active time [30], is sometimes targeted to wounds on the skin, and can reopen minor wounds (G. G. Carter 2013, personal observation) creating transmission potential. Allogrooming of the face and mouth is sometimes followed by regurgitations of ingested blood (e.g. [31]), which could also lead to RABV transmission [22]. No study has yet quantified changes in allogrooming in rabid bats.

During a study to evaluate a recombinant rabies vaccine candidate for vampire bats, we opportunistically measured rates of aggression and allogrooming in 40 captive male vampire bats that were experimentally infected with RABV. We then compared aggression and allogrooming in non-rabid bats to rabid bats confirmed RABV positive at death or at the end of the study.

## 2. Material and methods

### (a) Capture and care

We collected behaviour data from 40 male common vampire bats that were part of a larger sample of bats used to test a viral-vectored recombinant mosaic glycoprotein rabies vaccine candidate. We used males to reduce variability owing to reproductive status. The bats were captured in the State of San Luis Potosí, México, July–August 2018, and transported to the U.S. Geological Survey National Wildlife Health Center in Madison, Wisconsin, USA (for details see [21]). Bats were individually marked by combinations of 0–4 bat bands (Porzana Limited, Icklesham, UK) on the right or left forearm.

### (b) Experimental procedure

For the vaccination study, bats were initially caged according to three treatments: (i) oral vaccination, (ii) topical vaccination, or (iii) placebo control, and remained caged together for approximately 120 days before being challenged with RABV. One week prior to the challenge, we reassigned the bats into new groups, so individuals that received different treatments would be included in each cage and given time to acclimate. Each of the three cages (13–14 bats) had 3–5 bats per treatment. Group size was consistent with sizes of wild male vampire bat aggregations [17]. All bats were challenged with a heterologous RABV variant (of coyote origin) at a dose of 10^3.3^ tissue culture infective dose (TCID_50_ ml^−1^), injected intramuscularly into each masseter muscle (50 µl on each side) in April 2019 (127 days post-vaccination). We began quantifying behaviours one day after the challenge. To confirm death by rabies, we performed a direct fluorescent antibody test for RABV in brain impression smears of bats following standard procedures [32]. To detect RABV shedding in the saliva, we collected oral swabs periodically from all individuals, daily if clinical signs were observed, and upon death. Swabs were tested using real-time polymerase chain reaction as described elsewhere [33,34]. For further explanation of methods, see the electronic supplementary material.

### (c) Behavioural data collection

After bats were challenged with RABV, they were recorded using an infrared surveillance system (Amcrest 960 H/+) with a different camera pointed into each cage through a clear acrylic window. In each cage, we sampled behaviours 3 h per night (at hours 01.00, 03.00 and 05.00) during the most active period [35]. At every new-minute mark, an observer that was blind to the infection status of the bats stopped the video and recorded the presence or the absence of either allogrooming or aggression within a 5 s time window and the identities of the actor and receiver (using a unique combination of forearm bands). Allogrooming involves licking or chewing another bat's fur or skin and often occurs in both directions simultaneously (electronic supplementary material, video S1). Aggressive events included biting and fighting (electronic supplementary material, video S2), and a behaviour we call ‘clinging’ where a bat bites on to another's neck and clings onto it while the target tries to shake off the aggressor (electronic supplementary material, video S3).

### (d) Statistical analysis

Each night we collected 180 presence/absence samples per group except for two nights when 53 and 29 samples were lost owing to camera outages (resulting in a total of 18 818 behavioural samples). We counted the number of observed allogrooming and aggression events for each bat and divided it by the three sampled hours to estimate behavioural rates. We estimated 95% confidence intervals (CIs) around the mean rates for rabid and non-rabid bats using bootstrapping (percentile method, 5000 iterations, boot R package, [36]). To investigate if behavioural changes over time depended on infection status, we used a linear mixed effect model to test for an interaction between infection status and time (post-infection) with behaviour rate as the response and bat as a random intercept.

The exact timeline of infection was unclear before data collection. To determine whether the effect size was consistent across different possible time intervals before death, we plotted for every rabid bat the effect size during increasingly large (nested) time periods, from 1 day to 15 days before death (excluding one bat that survived until the end of the experiment 50 days after the challenge). To do this, we calculated the mean behaviour count for each focal rabid bat for a given period prior to death (e.g. days 1–4 prior to death), then compared that observed mean to the expected mean (i.e. the mean count of all non-rabid bats within the same group and time period). We calculated an effect size (Cohen's *d* [37]) for each time period:

$$\begin{array}{rl} & \mathrm{standardized}\;\mathrm{mean}\;\mathrm{difference}\\ & \;=\frac{\mathrm{mean}\;\mathrm{count}\;\mathrm{of}\;\mathrm{rabid}\;\mathrm{bat}-\mathrm{mean}\;\mathrm{count}\;\mathrm{of}\;\mathrm{non}-\mathrm{rabid}\;\mathrm{bats}}{\mathrm{pooled}\;\mathrm{standard}\;\mathrm{deviation}\;} \end{array}$$

## 3. Results

Fourteen of the 40 bats died after the experimental RABV challenge; all were confirmed RABV positive, and deaths occurred in all three cages and in all three treatment groups (five controls, four orally vaccinated, and five topically vaccinated). We did not detect a difference between the vaccination treatments on behavioural rates for either aggression or allogrooming in rabid bats (electronic supplementary material, figure S6, and table S7). The time of death ranged from 9 to 29 days post-challenge. One RABV-challenged bat that had been topically vaccinated was alive by the end of the experiment (after 50 days) but confirmed rabid by direct fluorescent antibody test. None of the 10 vaccinated bats that became rabid were shedding virus (assessed through real-time reverse transcription-polymerase chain reaction; see also [38]). Conversely, we detected RABV shedding in the saliva of 3 of 5 control bats on the day of death, and one of these was also shedding RABV the day prior to death.

**Figure 1.**
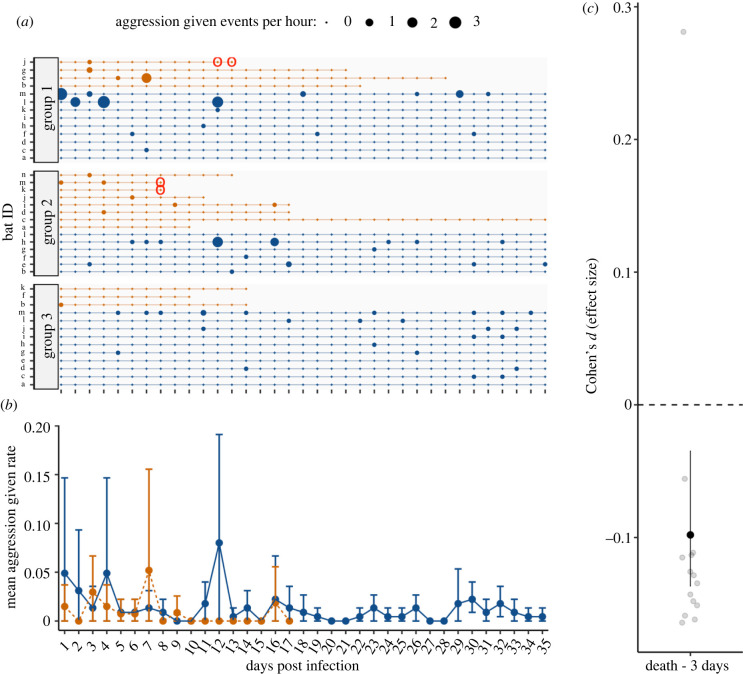
No evidence of increased aggression in rabid male vampire bats prior to death. Panel (*a*) shows timeline of aggression event counts for each rabid (orange points) and non-rabid (blue points) vampire bat across three groups. Point size reflects the rate of observed events per hour. Red circles show RABV positive saliva sample. Panel (*b*) shows mean rate of aggression events per hour with 95% CIs for rabid and non-rabid bats starting one day after inoculation with RABV. Panel (*c*) shows the standardized mean difference with 95% CIs between rabid bats and healthy cagemates in the three-day time interval before death. Outlier is one rabid bat (group 2-i) that showed high aggression 16 days post-challenge (2 days prior to death). See the electronic supplementary material, table S5 for CIs.

**Figure 2.**
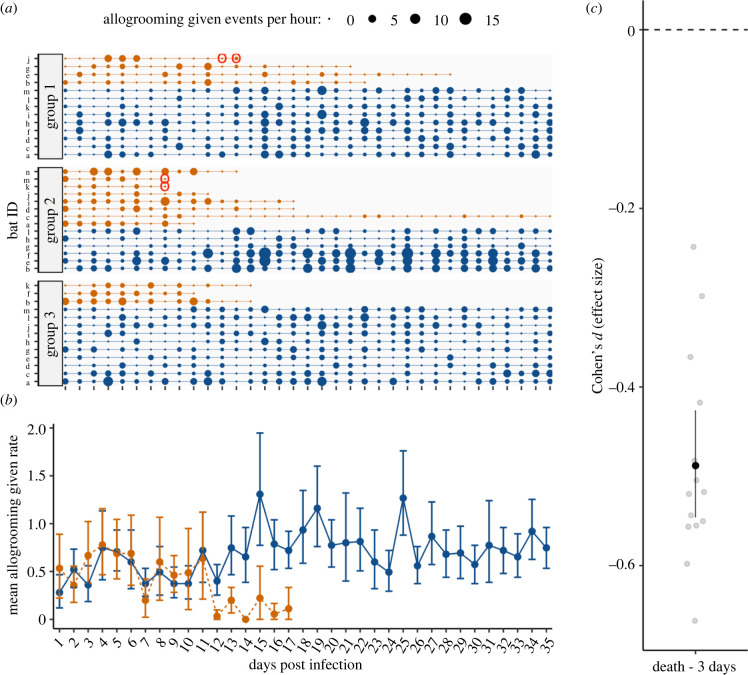
Reduced allogrooming prior to death in rabid male vampire bats. Panel (*a*) shows timeline of allogrooming event counts for each rabid (orange points) and non-rabid (blue points) vampire bat across three groups. Point size reflects the rate of observed events per hour. Red circles show RABV positive saliva sample. Panel (*b*) shows mean rate of allogrooming events per hour with 95% CIs for rabid and non-rabid bats starting 1 day after inoculation with RABV. Panel (*c*) shows the standardized mean difference with 95% CIs between rabid bats and healthy cagemates in the 3-day time interval before death. See the electronic supplementary material, table S5 for CIs.

In rabid bats, we did not detect any increase over time in aggression given (figure 1; interaction = −0.0009; *t* = −0.369_1192_; *p* = 0.7) or received (electronic supplementary material, figure S3; interaction = 0.0017; 0.783_1895_; *p* = 0.4). Instead, the effect of rabies status was negative and ‘small’ (*sensu* [37]), suggesting reduced aggression in rabid compared to healthy bats that did not clearly change with time (figure 1; electronic supplementary material, figures S2 and S3). Compared to their healthy cagemates, rabid vampire bats showed a reduction over time in allogrooming given (figure 2; interaction = −0.0295; *t* = −3.771_2319_; *p* < 0.005) and received (electronic supplementary material, figure S4; interaction = −0.0284; −3.704_2289_; *p* < 0.005). This ‘medium-sized’ effect (*sensu* [37]) occurred on average about 12 days after inoculation and increased as we considered time periods closer to their death (figure 2; electronic supplementary material, figures S2 and S4). The decrease in allogrooming and possible decrease in aggression before death are consistent with paralytic rather than furious rabies.

## 4. Discussion

Male vampire bats infected with a coyote RABV variant reduced their allogrooming to others, and probably as a consequence, also received less allogrooming. All bats showed low rates of aggression, and we saw no clear increase in aggression in the rabid bats, regardless of vaccine treatment group. Instead, aggression may have decreased along with general activity. Alternatively, because the effect size for aggression did not clearly amplify with time as with allogrooming, it is also at least possible that aggression was already lower in bats that became rabid. Taken together, these changes could be owing to either rabies-induced paralysis or generalized sickness behaviours, such as lethargy, causing passive self-isolation [39–42].

Compared to females, male vampire bats spend less time allogrooming in both the wild and captive groups [29,30]. However, males are probably more involved in the transmission of RABV between roosts [6–8] because they frequently move among roosts, disperse larger distances, and have more frequent aggressive interactions [18,19]. Current RABV transmission models benefit from considering the roles of sex and some infection-induced behavioural changes [7,8], but more empirical work could discern how behavioural effects vary with sex or impact transmission both within and between roosts.

Several other studies did not observe heightened aggression in rabid vampire bats (table 1). One possible reason for this lack of observations is reduced selection on RABV to increase aggression in vampire bats because they are highly social, frequently aggressive and bite other hosts [43]. Another possibility is that distinct RABV strains differ in pathogenicity and clinical forms of the disease (e.g. the presence of aggression) across species [44–47]. Studies describing natural infections often report some aggression, but experimental RABV challenges that failed to find evidence of aggression used viral strains that were not currently circulating or, as in our case, used a strain derived from a different species, not previously used in vampire bats, and thus the pathogenicity was unknown (table 1). It would be interesting to determine if infection with endemic vampire bat RABV strains may induce a higher proportion of furious versus paralytic disease.

Consistent with field observations [17,28], some rabid bats in our study may have received increased aggression prior to death (electronic supplementary material, figure S3, e.g. bat groups 1-j, 2-i, 2-d, 3-b). It would be interesting to examine further evidence for avoidance of, or aggression towards, infected individuals [42,48].

In the late stage of infection, RABV spreads to the salivary glands and is excreted in saliva [44]. Evidence of RABV shedding in vampire bats prior to or at the time of death has been demonstrated before [20,25,49]. Here, we detected RABV shedding in saliva of 3 of 15 rabid bats (all three unvaccinated), which allowed us to overlay behavioural measures with pathogen shedding (figures 1 and 2; electronic supplementary material, figures S3 and S4). These three vampire bats were not grooming others much when the virus was detectable in their saliva. Similarly, we did not observe heightened social aggression in these periods before death. Future work to quantify the relationship more closely between rabies shedding and behavioural changes would help clarify how these factors interact.

Times until death in rabid vampire bats varied from 9 to 29 days, but one of the 15 rabid males remained alive until the end of the experiment, 50 days after infection. The bat was previously vaccinated, but its neurologic function declined over the final weeks, losing coordination and mobility. We did not detect RABV in its saliva. The causes of this prolonged survival remain unclear.

One should consider several caveats when interpreting experimental results to date (table 1). First, given that aggression can be rare and brief, the absence of evidence of aggression is not evidence of absence. Aggression might have occurred at unsampled locations or times. For example, we observed anecdotal evidence of aggression by some rabid bats towards handlers and other bats when the bats were disturbed. Second, the administered RABV challenge dose, route and site of inoculation are not standardized across experiments (table 1). As in our study, the RABV challenge strains typically used for experimental infections are not currently endemic viruses, are derived from different species, or are adapted to other species. More standardized experimental infections are needed to disentangle the roles of administered dose and temporal overlap of circulating strains on rabies-induced behavioural changes in natural reservoirs such as vampire bats.

In conclusion, we observed reductions in allogrooming and low levels of aggression that indicated paralytic but not furious rabies presentation in 15 rabid male vampire bats relative to 25 non-rabid male bats. Alongside other previous reports involving natural rabies exposures that report elevated aggression (table 1), our results are consistent with the hypothesis that behavioural effects of RABV may vary by strain.

## Data Availability

All data and R code to repeat the analysis is publicly available on Figshare: https://doi.org/10.6084/m9.figshare.19991204.v4 [50]. Electronic supplementary material is accessible on Figshare [51].
